# Gerosuppression by pan-mTOR inhibitors

**DOI:** 10.18632/aging.101155

**Published:** 2016-12-30

**Authors:** Olga V. Leontieva, Mikhail V. Blagosklonny

**Affiliations:** ^1^ Department of Cell Stress Biology, Roswell Park Cancer Institute, Buffalo, NY 14263, USA

**Keywords:** dual mTORC1/C2 inhibitors, rapalogs, sirolimus, aging, cancer, senescence

## Abstract

Rapamycin slows organismal aging and delays age-related diseases, extending lifespan in numerous species. In cells, rapamycin and other rapalogs such as everolimus suppress geroconversion from quiescence to senescence. Rapamycin inhibits some, but not all, activities of mTOR. Recently we and others demonstrated that pan-mTOR inhibitors, known also as dual mTORC1/C2 inhibitors, suppress senescent phenotype. As a continuation of these studies, here we investigated in detail a panel of pan-mTOR inhibitors, to determine their optimal gerosuppressive concentrations. During geroconversion, cells become hypertrophic and flat, accumulate lysosomes (SA-beta-Gal staining) and lipids (Oil Red staining) and lose their re-proliferative potential (RPP). We determined optimal gerosuppressive concentrations: Torin1 (30 nM), Torin 2 (30 nM), AZD8055 (100 nM), PP242 (300 nM), both KU-006379 and GSK1059615 (1000 nM). These agents decreased senescence-associated hypertrophy with IC50s: 20, 18, 15, 200 and 400 nM, respectively. Preservation of RPP by pan-mTOR inhibitors was associated with inhibition of the pS6K/pS6 axis. Inhibition of rapamycin-insensitive functions of mTOR further contributed to anti-hypertrophic and cytostatic effects. Torin 1 and PP242 were more “rapamycin-like” than Torin 2 and AZD8055. Pan-mTOR inhibitors were superior to rapamycin in suppressing hypertrophy, senescent morphology, Oil Red O staining and in increasing so-called “chronological life span (CLS)”. We suggest that, at doses lower than anti-cancer concentrations, pan-mTOR inhibitors can be developed as anti-aging drugs.

## INTRODUCTION

Rapamycin slows down aging in yeast [1, 2], Drosophila [3-7], worm [8] and mice [9-30]. It also delays age-related diseases in a variety of species including humans [31-46]. Numerous studies have demonstrated life extension by rapamycin in rodent models of human diseases [9-48]. The maximal lifespan extension is dose-dependent [26, 42, 49]. One explanation is trivial: the higher the doses, the stronger inhibition of mTOR. There is another explanation: mTOR complex 1 (mTORC1) has different affinity for its substrates. For example, inhibition of phosphoryla-tion of S6K is achieved at low concentrations of rapamycin, whereas phosphorylation of 4EBP1 at T37/46 sites is insensitive to pharmacological concentrations of rapamycin [50-61]. Unlike rapalogs, ATP-competitive kinase inhibitors, also known as dual mTORC1/C2 or pan-mTOR inhibitors, directly inhibit the mTOR kinase in both mTORC1 and mTORC2 complexes [56, 59, 62-65].

In cell culture, induction of senescence requires two events: cell cycle arrest and mTOR-dependent gero-conversion from arrest to senescence [66-75]. In proliferating cells, mTOR is highly active, driving cellular mass growth. When the cell cycle gets arrested, then still active mTOR drives geroconversion: growth without division (hypertrophy) and a compensatory lysosomal hyperfunction (beta-Gal staining) [76]. So senescence can be caused by forced arrest in the presence of an active mTOR [76]. Senescent cells lose re-proliferative potential (RPP): the ability to regenerate cell culture after cell cycle arrest is lifted. Quiescence or reversible arrest, in contrast, is caused by deactivation of mTOR. When arrest is released, quiescent cells re-proliferate [66, 67].

In one cellular model of senescence (cells with IPTG-inducible p21), IPTG forces cell cycle arrest without affecting mTOR. During IPTG-induced arrest, the cells become hypertrophic, flat, SA-beta-Gal positive and lose RPP. When IPTG is washed out, such cells cannot resume proliferation. Loss of RPP is a simple quantitative test of geroconversion. Treatment with rapamycin during IPTG-induced arrest preserves RPP. When IPTG and rapamycin are washed out, cells re-proliferate [68-73, 77]. Recently, we have shown that Torin 1 and PP242 suppresses geroconversion, prevent-ing senescent morphology and loss of RPP [78, 79]. In agreement, reversal of senescent phenotype was shown by another pan-mTOR inhibitor, AZD8085 [80].

Pan-mTOR inhibitors have been developed as cytostatics to inhibit cancer cell proliferation. Cytostatic side effects in normal cells are generally acceptable for anti-cancer drugs. However, cytostatic side effects may not be acceptable for anti-aging drugs. Gerosuppressive (anti-aging) effects at drug concentrations that only mildly cytostatic are desirable. Pan-mTOR inhibitors differ by their affinity for mTOR complexes and other kinases. Here we studied 6 pan-mTOR inhibitors (in comparison with rapamycin) and investigated effects of 6 pan-mTOR inhibitors on rapamycin-sensitive and -insensitive activities of mTOR, cell proliferation and geroconversion.

## RESULTS

**Figure 1:**
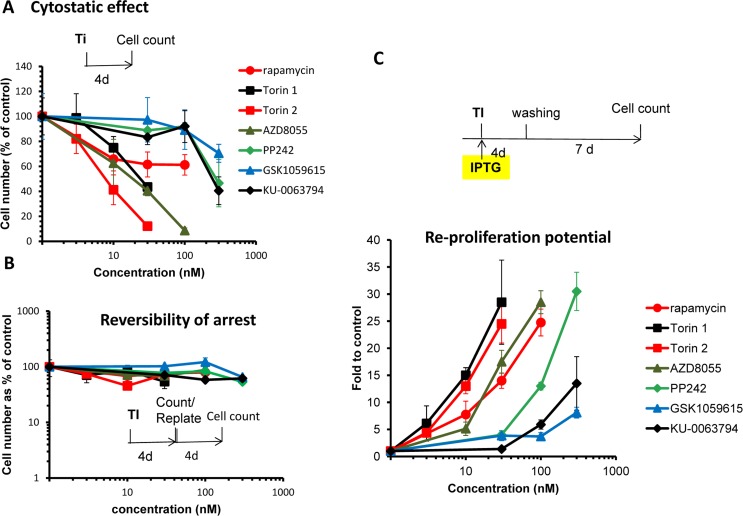
(A) Cytostatic effect. Effect of TOR inhibitors (Ti) on proliferation. HT-p21 cells were treated with serial dilutions of indicated Ti for 4 days and counted in triplicates. Data presented as mean ± SD. (B) Reversibility. Cells were treated as in A. After 4 day-treatment cells were counted and re-plated at 1000/well in 6-well plates in drug-free medium. Cells were allowed to re-proliferate for 4 days and counted. Cytostatic arrest was fully reversible. (C) Gerosuppression. Effect of TOR inhibitors on re-proliferative potential. HT-p21 cells were treated with IPTG in the presence of different concentrations of indicated Ti in triplicates. After 4 day-treatment, cells were washed off the drugs and allowed to regrow in drug-free medium for 7 days and counted. Data presented as mean ± SD

First we investigated the relationship between cytostatic and gerosuppressive activities of 6 pan-mTOR inhibitors: Torin1, Torin 2, AZD8055, PP242, KU-006379 and GSK1059615. All inhibitors inhibited proliferation in a dose-dependent manner (Fig. 1A). Inhibitory concentrations 50 (IC50) varied: Torin1 (22 nM), Torin 2 (8 nM), AZD8055 (20 nM), PP242 (285 nM), KU-006379 (230 nM) and GSK1059615 (>300 nM). At IC50, no cell death was observed. The inhibitory effect was cytostatic rather than cytotoxic and, further-more, reversible (Fig. 1B). When cells were treated with pan-mTOR inhibitors for 4 days and then re-plated and incubated in drug-free medium, the cells re-proliferated as efficiently as untreated control cells (Fig. 1B).

In the same cell line, HT-p21, we also measured gerosuppressive activities of mTOR inhibitors, by measuring re-proliferative potential (RPP) after induction of senescence with IPTG (Fig. 1C and Suppl. Fig. S1). In HT-p21 cells, IPTG induces p21, which in turn causes cell cycle arrest [76]. During cell cycle arrest, mTOR drives geroconversion to senescence, characterized by loss of RPP [68-73, 77]. Loss of RPP becomes evident after washing IPTG out. Although cells re-enter cell cycle, they cannot proliferate [81].

Inhibitors of mTOR preserved RPP in IPTG-treated cells. When IPTG and inhibitors of mTOR were washed out, the cells re-proliferated. By counting cell numbers after IPTG is washed out, we can measure gero-suppressive effects of mTOR inhibitors.

As shown in figure 1C and S1, all TOR inhibitors demonstrated equal maximal gerosuppressive activity, however, at different concentrations. Therefore, they have equal efficacy and different potency. (Note: Efficacy: maximum effect that mTOR inhibitor can cause regardless of concentration. Potency: concentration that is needed to cause this effect.) When we compared cytostatic versus gerosuppressive effects for each compound (Fig S2), we noticed that the gero-suppressive effect mirrored the cytostatic effect.

The lower concentration was required to inhibit proliferation, the lower concentration was required to suppress geroconversion (Suppl. Fig. S2). We estimat-ed concentration at which compounds exerted maximum gerosuppressive effect (Fig. 1C and Suppl. Fig. S1). Torins 1 and 2 turned out to be the most potent and GSK1059615 was the least potent. Torin 1 and 2 showed the same maximal effect in suppressing geroconversion at 30 nM (Fig.1C and Suppl. Fig. S1). Maximal gerosuppressive effect was achieved by GSK1059615 and KU-0063794 at 1000 nM (Suppl. Fig. S1). AZD8055 displayed maximum gerosuppressive effect at 100 nM. As seen in figure S1, gerosuppressive effects reached the plateau and then decreased at higher concentrations, due to toxicity.

### Preservation of RPP correlated with inhibition of mTORC1

MTOR complex 1 (mTORC1) phosphorylates S6 kinase (S6K) at T389, which in turn phosphorylates S6 at S235/236 and S240/244. This S6K/S6 axis is rapamycin-sensitive. Phosphorylation of 4EBP1 at T37/46 is rapamycin-insensitive. Function of mTORC2, which is rapamycin-insensitive, can be measured by phospho-AKT (S473), albeit it is not the only kinase that phosphorylates Akt at that site.

**Figure 2:**
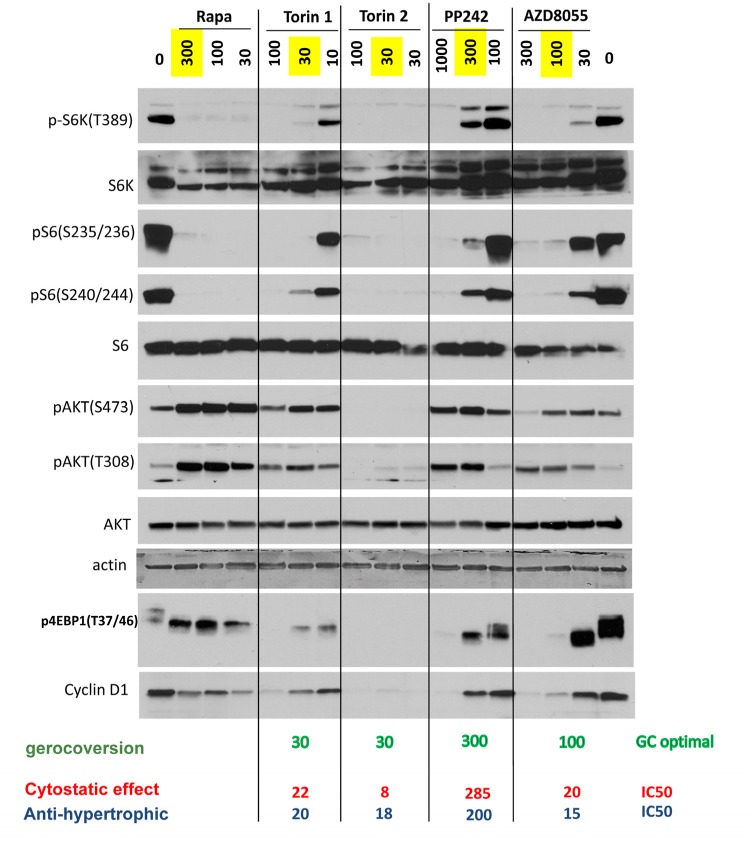
Effect of TOR inhibitors on mTOR-pathway in HT-p21 cells Cells were treated with IPTG and different concentrations of indicated inhibitors for 24h and lysed. Immunoblotting was performed with indicated antibodies. Maximal optimal gerosuppressive concentrations are highlighted in yellow.

At optimal gerosuppressive concentrations, pan-mTOR inhibitors decreased phosphorylation of S6K at T389 (target of mTORC1) and its downstream targets S6 (S235/236) and (S240/244) (Fig. 2 and Suppl. Fig. S3). At optimal concentration (30 nM), Torin 2 inhibited phosphorylation of AKT at S473 and T308. Other inhibitors, at optimal gerosuppressive concentrations, did not decrease phosphorylation of AKT or even caused an increase in level of pAKT(S473) and/or pAKT(T308) similar to the effect of rapamycin, which induces phosphorylation of AKT in HT-p21 cells (Fig. 2). We conclude that mTORC2 and/or AKT in particular are not essential for geroconversion, as measured by RPP, in HT-p21 cells. Phosphorylation status of 4EBP1, a substrate of TORC1, was revealing. Rapamycin caused mobility shift but did not inhibit phosphorylation at the particular T37/46 sites. Torin 2 inhibited 4EBP1 phosphorylation at T37/46 sites. At optimal gerosuppressive concentrations, all other pan-mTOR inhibitors caused mobility shift and only marginally decreased T37/46 phosphorylation, which however was inhibited at higher concentrations (Fig. 2 and Suppl. Fig. S3).

### Pan-mTOR inhibitors prevent cellular hypertrophy

**Figure 3:**
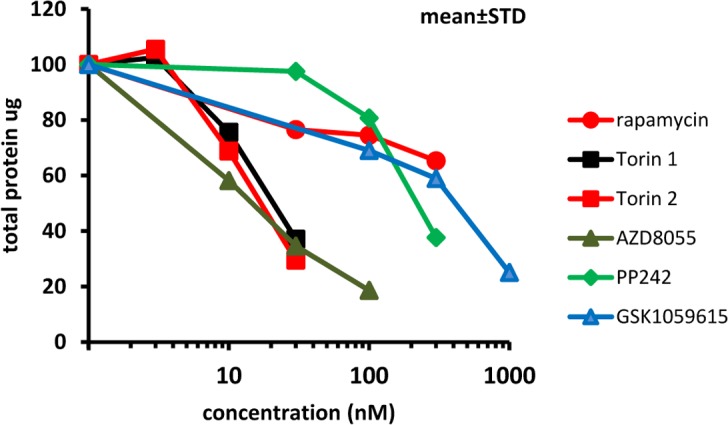
Effect of TORINs on protein level in senescent HT-p21 cells Cells were treated with IPTG and different concentrations of TORINs for 4 days and protein amounts were measured. Data are mean ± SD.

We next determined effects of mTOR inhibitors on senescence-associated hypertrophy in IPTG-arrested HT-p21 cells. Hypertrophy can be measured as protein per cell [82]. IPTG induces cell cycle arrest, so that cells do not proliferate and the number of plated cells stays the same throughout the treatment [82]. Therefore, hypertrophy can be easily determined by measuring protein per well. We treated cells with IPTG and its combination with mTOR inhibitors. After a 4 day-treatment, cells were lysed and protein was measured. Pan-mTOR inhibitors decreased cellular hypertrophy in a dose-dependent manner. Rapamycin was an exception, i.e. its inhibitory effect on cellular hyper-trophy was moderate and reached a plateau. IC50 values were as follows: 20, 18, 15, 200 and 400 nM for Torin 1, Torin 2, AZD8085, PP242 and GSK1059615, respectively (Fig. 3). All inhibitors reduced amount of protein by more than 50% at concentrations corresponding to their optimal gerosuppressive concentrations measured by RPP.

**Figure 4:**
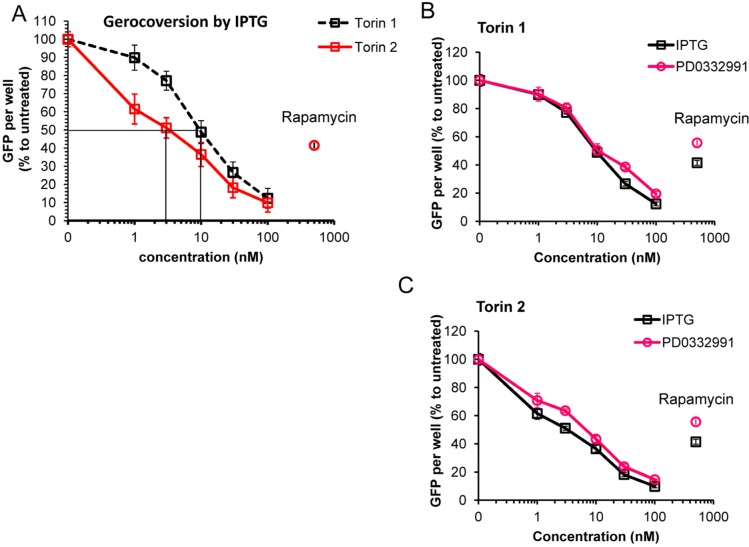
Effect of torins 1 and 2 on hypertrophy of senescent HT-p21 cells measured by constitutive GFP fluorescence of these cells (**A**) HT-p21 cells were treated with IPTG and concentration range of torin1 or torin 2, rapamycin (500 nM) was included for comparison as additional control. After 4 day-treatment GFP fluorescence was quantified using Typhoon scanner (Amersham Biosciences variable mode imager) and ImageQuantTL software. (**B**) and (**C**) HT-p21 cells were induce to senesce by treatment with either IPTG (3 days) or PD0332991 (0.5 μM, for 4 days) and concentration range of torin 1 (**B**) or torin 2 (**C**). Effect of torins on hypertrophy was assessed by measuring GFP fluorescence as described in (**A**). GFP per well is presented as % to IPTG or PD0332991 only treated cells for each set. Data are means ± SE of 8 replicates from one out of three independent experiments.

We next employed additional method of measuring cellular hypertrophy by measuring GFP under CMV-constitutive promoter in HT-p21 cells. (HT-p21 cells are stably transfected with GFP-CMV). It was previously shown that GFP accumulation is a marker of hypertrophy [82]. Torin 2 was more potent anti-hypertrophic agent than Torin 1 (Fig. 4A). IC50 values were 3 nM and 10 nM for torn 1 and 2, respectively (Fig. 4A). At 30 nM, both Torins were more anti-hypertrophic than rapamycin (Fig. 4A, rapamycin was used at 500 nM). Anti-hypertrophic effect of Torins was independent of the nature of senescence-inducing agent, i.e. IPTG-inducible ectopic p21 or inhibitor of CDK4/6 PD0332991 (Fig. 4B, C). We conclude that Torins blocked senescence-associated hypertrophy more effectively compared with rapamycin. Further-more, Torin 1, which is more rapamycin-like than Torin 2, was less potent as anti-hypertrophic agent than Torin 2.

### Torins 1 and 2 decrease lipid accumulation in senescing cells

**Figure 5:**
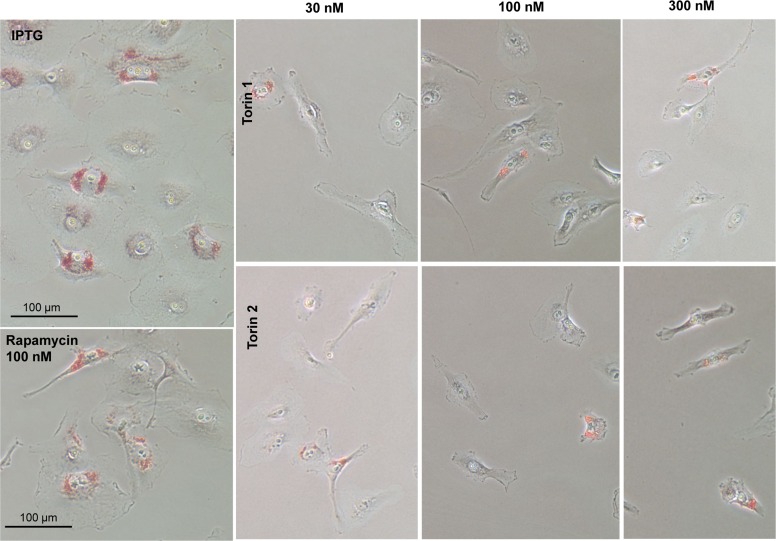
Effect of torin analogs on lipid accumulation in senescent HT-p21 cells Cells were treated with IPTG and concentrations range of torin 1or torin 2 for 4 days and stained with Oil Red O. Bar – 100 μm.

One of the features of senescent HT-p21 cells is accumulation of lipids, which is detected as positive Oil Red O staining in perinuclear region (Fig. 5, IPTG). When these cells were co-treated with IPTG and Torins 1 or 2, cells remained small and Oil Red O negative (Fig. 5). As in the case of SA-beta-Gal staining, rapamycin was less effective than Torins in decreasing this marker of senescent HT-p21 cells.

### Pan-mTOR inhibitors prolongs CLS in HT-p21 cells

**Figure 6:**
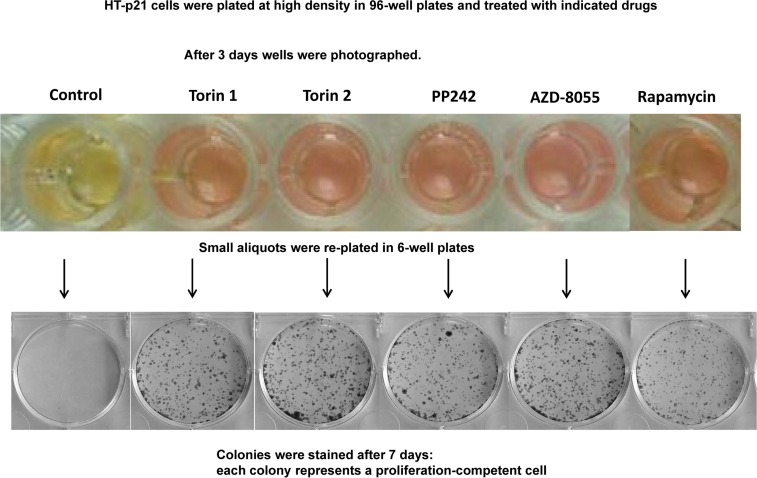
Effect of TOR inhibitors on chronological senescence of cancer HT-p21 cells Cells were plated at high density in 96-well plates and treated with TOR inhibitors or rapamycin at selected optimal concentrations. After 3 days in culture cells were photographed (color manifests pH of medium), trypsinized and small aliquots were re-plated in 6-well plates. Formed colonies were stained after 7 days in culture with Crystal Violet.

The yeast is commonly used as a model of aging. In particular, rapamycin extends chronological lifespan (CLS) [2]. In stationary culture, yeast cells lose viability measured as re-proliferative potential in fresh culture [1-7]. It is erroneously believed that “chronological aging” is an equivalent of aging of post-mitotic cells in multicellular organism. In reality, this phenomenon is an equivalent of lose of cancer cell viability in overcrowded culture [83]. Both yeast and cancer cells acidify the culture medium and lose viability, as measured for example by re-proliferation in fresh low-density culture. When plated at very high cell density, HT-p21 cells produce high levels of lactic acid, acidifying medium (“yellow color”). This causes loss of re-proliferative potential [83]. Rapamycin extends CLS by decreasing lactate production [83]. Here we tested whether pan-mTOR inhibitors can extend CLS of HT-p21 cells. After 3 days in a high-density culture, HT-p21 cells remained alive, but could not re-proliferate and form colonies when re-plated in fresh medium (Fig. 6, control). When high-density cultures were treated with mTOR inhibitors, these cells produced less lactic acid as seen by the color of the medium (Fig. 6; yellow indicates low pH and high levels of lactic acid) [84]. They maintained re-proliferative potential and formed colonies when re-plated in fresh medium in low density (Fig. 6). Rapamycin was less effective than pan-mTOR inhibitors. At equipotent (optimal) concentrations, all pan-mTOR inhibitors showed similar efficacy in prolonging chronological life span.

### Pan-mTOR inhibitors suppress senescent morphology of SKBR3 and MEL10 cells

**Figure 7:**
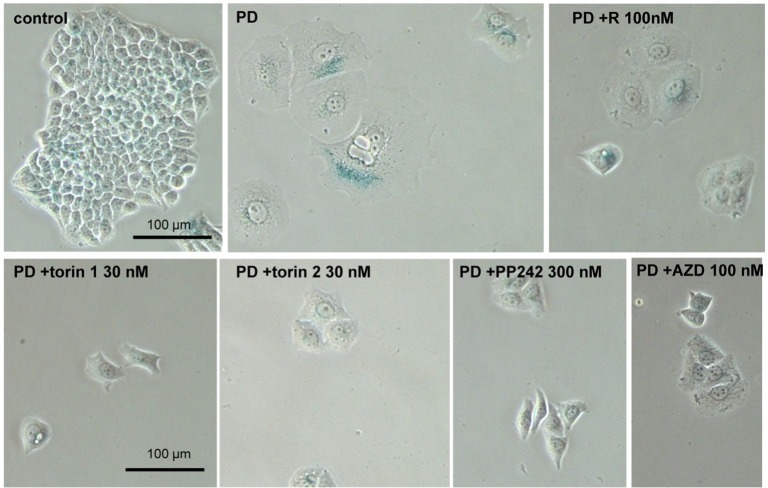
Effect of TORINs on senescent morphology of SKBR3 cells induced to senesce by treatment with PD0332991 Cells were treated with selected concentrations of TORINs and 10 μM PD0332991 (PD). After 4-day treatment drugs were washed out and cells were incubated in drug free medium for 2 days and stained for SA-beta-gal. Bar – 100 μm.

**Figure 8:**
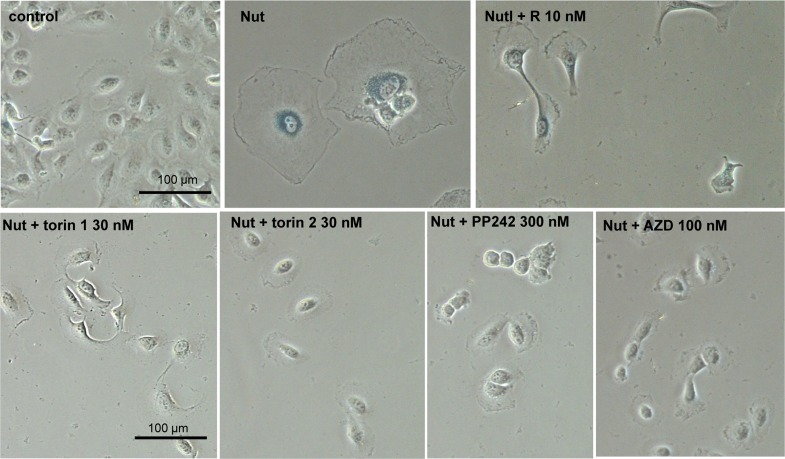
Effect of TORINs on senescent morphology of MEL10 cells undergoing senescence by treatment with nutlin 3a Cells were treated with nutlin 3a (2.5 μM) and TORINs at selected concentrations or rapamycin (R). After 4‐day treatment drugs were washed out and cells were incubated in drug‐free medium for another 2 days and stained for SA‐beta‐gal. Bar – 100 μm. Nut – nutlin 3a; AZD – AZD8085.

We next investigated gerosupressive effects of mTOR inhibitors in SKBR3 and MEL10 cells undergoing geroconversion after treatment with CDK4/6 inhibitor PD0332991 and nutlin-3a, respectively **[72]**. As shown in Fig 7, treatment with PD0332991 caused senescent morphology in SKBR3 cells. Co-treatment with pan-mTOR inhibitors prevented senescent morphology and hypertrophy (Fig.7 and Suppl. Fig. S4A). Pan-mTOR inhibitors also prevented senescent morphology of MEL10 cells induced to senesce by treatment with low concentration of nutlin-3a (Fig. 8 and Suppl. Fig. S4B).

## DISCUSSION

As predicted by theory of TOR-driven aging [29, 85-97], rapamycin extends life span and prevents age-related diseases (see Introduction). Yet, rapamycin (and other rapalogs such as everolimus) does not inhibit all functions of mTOR. Inhibition of both rapamycin-sensitive and --insensitive functions of mTOR may be translated in superior anti-aging effects. However, potential benefits may be limited by undesirable effects such as inhibition of cell proliferation (cytostatic effect) and cell death (cytotoxic effect). In fact, pan-mTOR inhibitors have been developed to treat cancer, so they are cytostatic and cytotoxic at intended anti-cancer concentrations. Yet, the window between gero-supressive and cytotoxic effects exists. At optimal gerosuppressive concentrations, pan-mTOR inhibitors caused only mild cytostatic effect. For Torin 1 and PP242, the ratio of gerosuppressive (measured by RPP) to cytostatic concentrations was the most favorable. The ratio of anti-hypertrophic to cytostatic concentration was similar for all pan-mTOR inhibitors.

Gerosuppressive effect of pan-mTOR inhibitors (as measured by RPP) was equal to that of rapamycin because it is mostly associated with inhibition of the S6K/S6 axis. Yet anti-hypertrophic effect as well as prevention of SA-beta-Gal staining and large cell morphology was more pronounced with pan-mTOR inhibitors than with rapamycin. Also, at optimal concentrations, all pan-mTOR inhibitors extended loss of re-proliferative potential in stationary cell culture more potently than rapamycin. This test determines hyper-metabolism and lactic acid production and is an equivalent of “yeast CLS” (see [83]. One conclusion is that pan-mTOR inhibitors may be superior to rapamycin.

At low concentrations, pan-mTOR inhibitors acted like rapamycin, inhibiting the S6K/S6 axis and causing mobility shift of 4EBP1 (Fig. 2). With increasing concentrations, these drugs inhibited phospho-4EBP1 (T37/46) followed by inhibition of phospho-AKT (S473) (Fig. 2) and thereby further contributed to anti-hypertrophic effects (and cytostatic effect), prevention of senescent morphology as well as inhibition of CLS. Importantly, effects of pan-mTOR inhibitors varied in their resemblance to rapamycin effects. In particular, Torin 1 and PP242 were rapamycin-like. The window between inhibition of pS6K/S6 versus p4EBP1 and AKT was narrower for Torin 2 and AZD8085 than for other 4 pan-mTOR inhibitors. In general, maximal gerosuppression (as measured by RPP) was achieved at concentrations that inhibited phosphorylation of S6K and S6 and only partially inhibited rapamycin-insensitive functions of mTOR. Rapamycin-like effects achieved at lower concentrations of pan-mTOR inhibitors than rapamycin–unlike effects. Preservation of RPP depends on rapamycin-sensitive functions. Inhibition of senescent morphology (SA-beta-Gal staining, hypertrophy, flat morphology) and CLS depends on both rapamycin-sensitive and -insensitive functions of mTOR.

At gerosuppressive concentrations, pan-mTOR inhibitors should be tested as anti-aging drugs. Life-long administration of pan-mTOR inhibitors to mice will take several years. Yet, administration of pan-mTOR inhibitors can be started late in life, thus shortening the experiment. In fact, rapamycin is effective when started late in life in mice [9]. Optimal doses and schedules of administration could be selected by administration of pan-mTOR inhibitors to prevent obesity in mice on high fat diet (HFD). It was shown that high doses of rapamycin prevented obesity in mice on HFD even when administrated intermittently [21, 98-100]. Testing anti-obesity effects of pan-mTOR inhibitors will allow investigators to determine their effective doses and schedules within several months. It would be important to test both rapamycin-like agents such as Torin 1 and rapamycin-unlike agent such as Torin 2 or AZD8085. Selected doses and schedules can then be used to extend life-span in both short-lived mice, normal and heterogeneous mice as well as mice on high fat diet. These experiments will address questions of theoretical and practical importance: (a) role of rapamycin-insensitive functions of mTOR in aging. We would learn more about aging and age-related diseases. (b) can pan-mTOR inhibitors extend life span beyond the limits achievable by rapamycin. If successful, such experiments may reveal new causes of death in the absence of mTOR-driven aging, a post-aging syndrome, as mentioned previously [101]. Given that pan-mTOR inhibitors are already undergoing clinical trials for cancer therapy, one can envision their fast application for prevention of age-related diseases by slowing down aging.

## MATERIALS AND METHODS

### Cell lines and reagents

HT-p21 cells, derived from human fibrosarcoma HT1080, were described previously [69, 76, 81, 102, 103]. In HT-p21 cells, p21 expression can be turned on and off using IPTG (isopropyl-thio-galactosidase). These cells express GFP under CMV promoter. HT-p21 cells were cultured in DMEM/10% FC2 serum (HyClone FetaClone II; Thermo Scientific). Melanoma cell line MEL10 and breast adenocarcinoma SKBR3 (ATCC, Manassas, VA) were maintained in DMEM/10% FBS.

Rapamycin was purchased from LC Laboratories (Woburn, MA). Pan-mTOR inhibitors (torin 1, torin 2, PP242, AZD8085, KU-0063794, GSK1059615) and PD0332991 were from Selleckchem (Houston, TX). Stock solutions were prepared in DMSO.

### Re-proliferative potential (RPP)

HT-p21 cells were plated at low densities and treated with IPTG alone or in combination with mTOR inhibitors as described in figure legends. After 3-4 days, IPTG and drugs were washed out and cells were allowed to re-proliferate in drug free medium for 7 days and counted in triplicates.

### Immunoblot analysis

Cells were lysed using boiling lysis buffer (1% SDS, 10 mM Tris.HCl, pH7.4). Protein concentrations were measured using BCA protein reagent (Thermo Scientific) and equal amounts of protein were separated on 10% or gradient polyacrylamide gels and transferred onto PVDF membranes [69, 81]. The following antibodies were used: rabbit antibodies against phospho-pS6K(T389), phospho-S6(S235/236) and phospho-S6(S240/244), S6K, phospho-4EBP1(T37/46), phospho-AKT(S473) and phospho-AKT(T308), AKT and mouse anti-S6 – from Cell Signaling Technology (Danvers, MA); mouse monoclonal antibodies against cyclin D1 and rabbit anti-actin were from Santa Cruz Biotechnology (Paso Robles, CA) and Sigma-Aldrich (St. Louis, MO), respectively.

### SA-β-galactosidase staining

β-gal staining was performed using Senescence-galactosidase staining kit from Cell Signaling Technology according to manufacturer's protocol. Cells were microphotographed under light microscope [69, 73].

### CLS in mammalian cells

Cells were plated at high density in 96-well plates. After 3 days, cells were trypsinized and a small aliquot of attached cells was re-plated at low density in 6-well plates in fresh medium. After 7 days in culture colonies were stained with 1% Crystal Violet (Sigma-Aldrich) [83].

### Oil Red O staining

0.35% Oil Red O (Sigma-Aldrich) stock was prepared in isopropanol. Working solution was prepared fresh before use by mixing 3 parts of Oil Red O stock with 2 parts of water and incubating it at RT for 20 min followed by filtering through 0.2 μm filter. Cells were washed with PBS and incubated in 10% formalin at RT for 10 min and then with refreshed formalin for another 1 h followed by two washes in ddH_2_O. Fixed cells were incubated in 60% isopropanol for 5 min at RT followed by incubation with working solution of Oil Red O for 20 min. After extensive washes in ddH_2_O, cells were microphotographed under light microscope.

## SUPPLEMENTARY MATERIAL FIGURES


