# Supplementary material for: Gerosuppression by pan-mTOR inhibitors
Source: Aging (Albany NY). 2016 Dec 30;8(12):3535–49. doi: 10.18632/aging.101155 (PMC5270685; doi:10.18632/aging.101155)
Supplement: Supplementary file 1 [file aging-08-3535-s001.pdf]

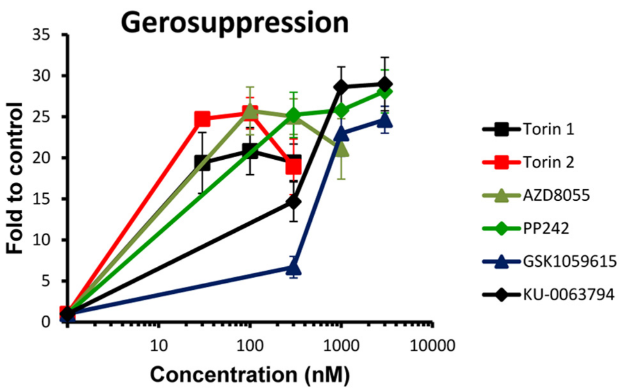

Supplementary Figure S1. Extended concentration ranges of TOR inhibitors to determine maximal optimal dose for gerosuppression in HT-p21 cellular model of senescence. HT-p21 cells were treated with IPTG and different concentrations of indicated TOR inhibitors. After 4 day-treatment, drugs were washed out and cells were incubated in drug-free medium For 7 days and counted. Data are mean ± SD from triplicate wells.

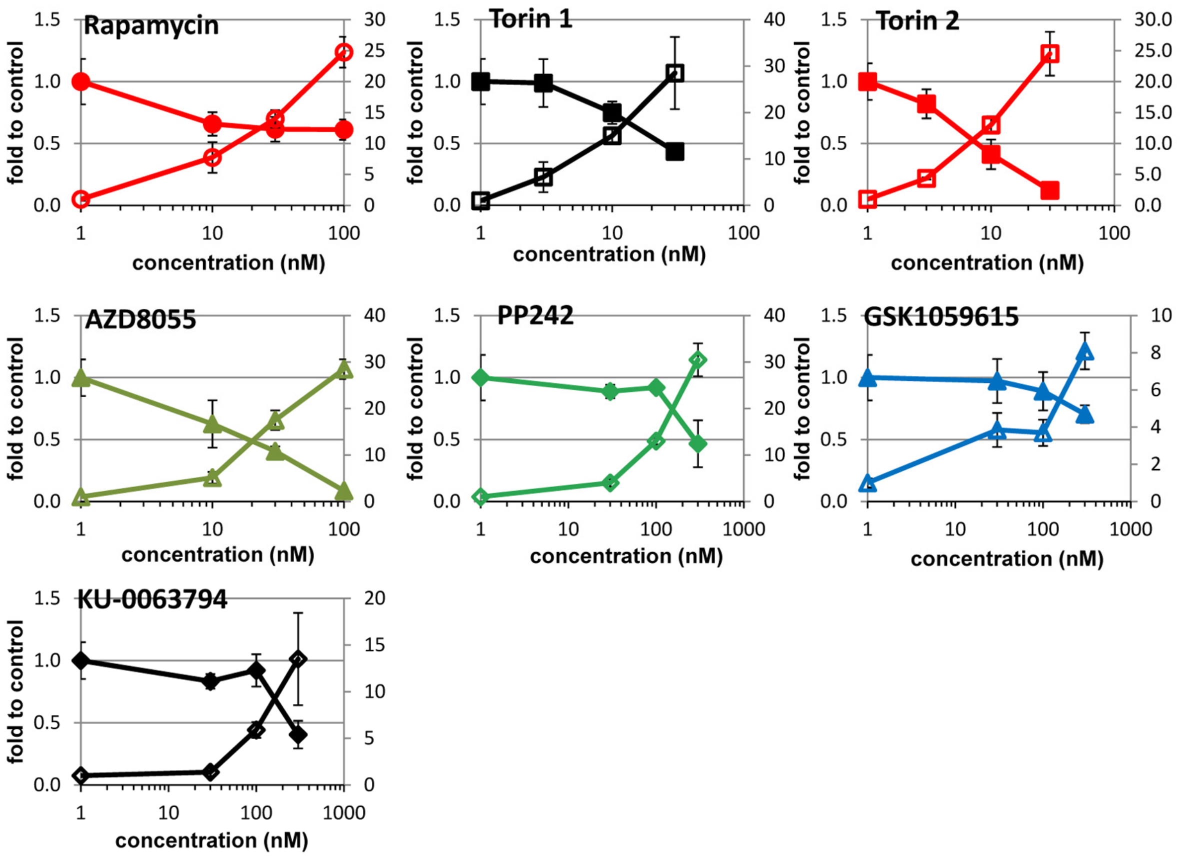

Supplementary Figure S2. Gerosuppressive effect mirrors cytostatic effect. of TOR inhibitors**.** HT-p21 cells were treated with serial dilutions of indicated drugs as described in Figure 1 A (for cytostatic effect, shown as filled markers) and in Fig. 1C (for gerosuppressive effect, shown as empty markers).

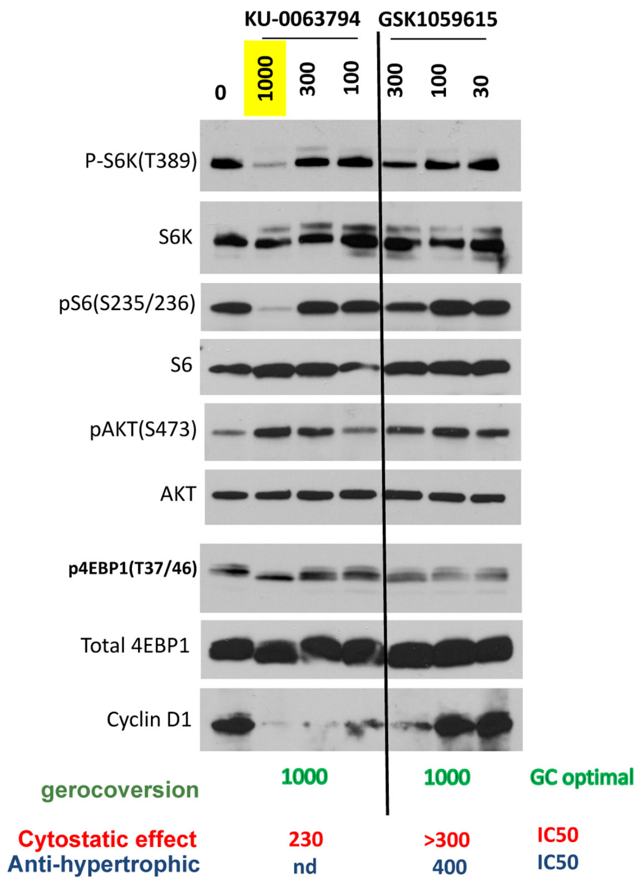

Supplementary Figure S3. HT-p21 cells were treated with range of concentrations of KU-0063794 and GSK1059615 for 24 h and lysed. Data present Immunoblotting with indicated antibodies.

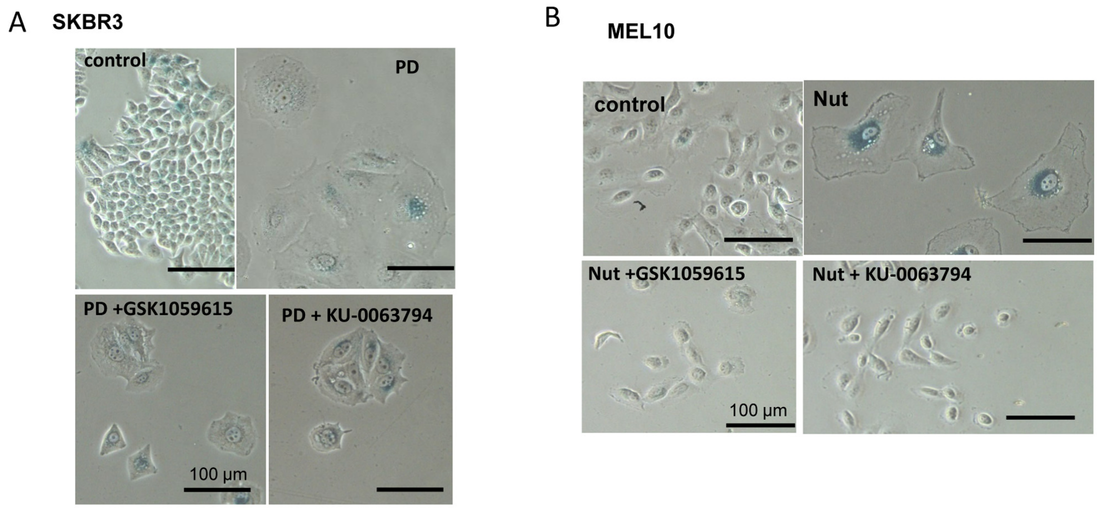

Supplementary Figure S4. Effect of GSK1059615 and KU-0063794 on senescent morphology of SKBR3 (A) and MEL10 (B) cells. SKBR3 and MEL10 cells were induced to senesce by treatment with 10 µM PD0332991 (PD) or 2.5 µM nutlin 3a (Nut), respectively. Co-treatment with either 1000 nM of GSK1059615 or KU-0063794 prevent senescent morphology in these cells.
